# Canaloplasty and Trabeculotomy Combined With Phacoemulsification for Primary Angle-Closure Glaucoma: A Single-Surgeon Case Series

**DOI:** 10.7759/cureus.60549

**Published:** 2024-05-18

**Authors:** Anna Mueller, Claire E Malley, Shannan Berzack, Rachel Israilevich, Juan Ruiz-Pelaez, Matthew Brink

**Affiliations:** 1 Department of Ophthalmology, Florida International University, Herbert Wertheim College of Medicine, Miami, USA; 2 Department of Ophthalmology, Mayo Clinic, Miami, USA; 3 Department of Translational Medicine, Florida International University, Herbert Wertheim College of Medicine, Miami, USA

**Keywords:** phacoemulsification, trabeculotomy, canaloplasty, anterior chamber angle, chronic angle closure glaucoma

## Abstract

Purpose: To evaluate the outcomes of combined canaloplasty and trabeculotomy with phacoemulsification for primary angle-closure glaucoma (PACG).

Methods: In this retrospective, consecutive, single-surgeon case series, we analyzed the pre- and postoperative measurements of PACG patients who had the procedure. Adverse events were recorded. The main outcomes were mean intraocular pressure (IOP) in each quartile of the follow-up year and the number of IOP-lowering medications the patients were on by the end of each quartile compared to their baseline values.

Results: A total of 46 eyes from 39 PACG patients were included. The preoperative IOP and glaucoma medications taken were 19.33±6.03 mm Hg and 1.80±1.39, respectively (N=46). Postoperative IOP means (mm Hg) in the subsequent four quartiles were 14.00±3.33 (N=44), 13.44±2.83 (N=32), 14.38±2.39 (N=16), and 14.92±2.90 (N=13) (p<0.0001). The mean number of meds was 0.32±0.80, 0.22±0.42, 0.59±0.80, and 0.08±0.28 in each respective quartile (p<0.0001), while the median was 0 across all quartiles.

Conclusions: Combining the OMNI surgical system with phacoemulsification led to substantial reductions in mean IOP and the number of IOP-lowering medications when compared to baseline measurements.

## Introduction

Primary angle-closure glaucoma (PACG) is a prevalent cause of irreversible blindness, accounting for nearly half of all glaucoma-related blindness cases worldwide [1]. PACG is associated with a threefold increase in the risk of severe bilateral vision loss when compared to POAG [2]. This multifactorial condition arises from anatomical crowding of the anterior chamber, characterized by iridotrabecular contact and angle closure. Impaired aqueous humor drainage results in trabecular meshwork (TM) and Schlemm’s canal (SC) degeneration, leading to progressive elevation of intraocular pressure (IOP) and the eventual development of glaucomatous optic neuropathy.

The management of PACG includes a range of interventions, including IOP-lowering drugs, laser iridotomy, laser iridoplasty, phacoemulsification, trabeculectomy, and microinvasive glaucoma surgery (MIGS) [3]. Of the surgical procedures, phacoemulsification, with or without goniosynechialysis (GSL), has demonstrated notable efficacy in reducing IOP and medication dependency in patients with PACG [4,5]. Phacoemulsification addresses the mechanical obstruction in PACG by relieving pupillary block, deepening the anterior chamber, and reducing the risk of peripheral anterior synechiae (PAS) formation [6]. However, addressing the mechanical obstruction in PACG may not be enough. Studies have shown that persistent iridotrabecular contact and PAS can damage the TM and SC through various mechanisms. These mechanisms include disrupted TM metabolism due to blood-aqueous barrier dysfunction, TM mitochondrial dysfunction resulting in fibrotic changes, SC endothelial damage leading to canal diameter reduction, and mechanical blockage by pigmentary granules and TM cells [7]. For this reason, glaucoma may persist following laser iridotomy or phacoemulsification [7]. Therefore, addressing the damage to the TM and SC from chronic disease may offer more favorable outcomes.

Trabeculectomy, with or without phacoemulsification, has been the standard-of-care surgical approach for PACG management for decades [1]. However, trabeculectomy presents challenges in postoperative care and carries significant risks, including suprachoroidal hemorrhage, choroidal detachment, chronic hypotony, bleb leaks, and endophthalmitis [8]. IOP reductions are smaller when compared to traditional trabeculectomy, but MIGS procedures offer a more favorable safety profile [9].

Canaloplasty combined with trabeculotomy using the OMNI® surgical system (Sight Sciences, Menlo Park, United States) has emerged as a promising MIGS procedure for PACG patients. This approach addresses three sources of aqueous humor drainage resistance: TM, SC, and collector channels [10]. The procedure involves micro-catheterization and transluminal viscodilation of SC through canaloplasty, followed by trabeculotomy. By simultaneously targeting these resistance points and coupling this with phacoemulsification, the combined procedure holds the potential to significantly reduce IOP and medication burden in PACG patients, targeting damaged TM and SC from long-standing disease. Despite its FDA clearance for IOP reduction in primary open-angle glaucoma, the utilization of this approach in PACG patients remains off-label, with no published data to date on its efficacy and safety profile. This study aims to describe the outcomes of canaloplasty and trabeculotomy with the OMNI system combined with phacoemulsification in patients with PACG.

## Materials and methods

This retrospective study was approved by the Institutional Review Board at Florida International University (IRB-22-0285) and is in compliance with the Declaration of Helsinki. Health Insurance Portability and Accountability Act (HIPAA) regulations were followed at all stages of the study. All patients diagnosed with PACG who underwent canaloplasty and trabeculotomy with the OMNI combined with cataract surgery between October 2021 and December 2022 in a private practice in Miami, United States, were identified from our electronic medical system. The diagnosis of angle closure was made when more than 180 degrees of appositional angle closure was present. A manual chart review was used to confirm patients had undergone combined canaloplasty and trabeculotomy with phacoemulsification.

Surgical approach

A Swan-Jacob lens was used intraoperatively to examine the angle and determine the best location for the canaloplasty/goniotomy. The OMNI cannula was used to incise the inner wall of SC nasally, advanced 180 degrees superonasally, and then retracted to complete the canaloplasty superiorly with ProVisc. The OMNI device was subsequently rotated 180 degrees, and the cannula was reinserted inferonasally into SC. The cannula was then advanced 180 degrees inferonasally and retracted to complete the canaloplasty with ProVisc. The cannula was again advanced into the inferonasal SC at 180 degrees, and the extended cannula was used to perform goniotomy at 180 degrees inferonasally. The ProVisc (Alcon Laboratories, Inc., Fort Worth, United States) and VisCoat (Bausch + Lomb, Bridgewater, United States) were then irrigated and aspirated out.

Clinical metrics

Patient demographic information, biometric measurements (axial length, anterior chamber depth, and lens thickness), lens status and cataract stage, relevant medical and surgical history, IOP measurements, number of IOP-lowering drugs taken, best corrected visual acuity (BCVA) and adverse events intra- and postoperatively were recorded. All IOP readings were obtained by a single surgeon using Goldmann applanation tonometry. The most recent preoperative IOP and IOP-lowering medication counts were recorded. Postoperative quartiles were defined as three-month periods following surgery. IOP measurements from the first postoperative week were excluded from the first quartile calculation. Adverse events were recorded intra-operatively, immediately following surgery, and through the final follow-up visit. Non-responders were defined as patients with elevated IOP exceeding 21 mm Hg on at least two follow-ups, those requiring two or more postoperative IOP-lowering medications, and/or patients maintaining their preoperative medication count.

Statistical analysis

Stata Version 17 (Stata Corp LLC, College Station, United States) and GraphPad Prism 10 (GraphPad Software Inc., San Diego, United States) were used for data analyses and visualization. Each quartile outcome was compared to preoperative values using a paired t-test. Longitudinal changes were examined via mixed-effects analysis. A p-value less than 0.05 was considered statistically significant.

## Results

A total of 46 eyes from 39 PACG patients were eligible for study inclusion. All patients were phakic and had visually significant cataracts. Most patients had no trabecular meshwork visible on gonioscopy throughout 360 degrees of the angle. Synechial angle closure and extensive PAS (more than one clock hour) were rare. Demographics and baseline characteristics are described in Table 1. In brief, the mean patient age was 73.0±8.5, with 60.9% being female and the largest racial/ethnic group being Hispanic (41.3%). Notably, 54.3% of the subjects had systemic hypertension, and 28.3% had diabetes mellitus. The mean axial length, anterior chamber depth, and lens thickness were 23.3±0.9 mm, 2.8±0.30 mm, and 4.9±0.4 mm, respectively. Prior interventions included laser peripheral iridotomy (13.0%) and selective laser trabeculoplasty (15.2%). The baseline BCVA in LogMar was 0.4±0.4. The baseline IOP averaged 19.3±6.0 mm Hg, with a median of 18.5 mm Hg. The mean number of preoperative IOP-lowering agents was 1.8±1.4, with a median of two IOP-lowering drops.

**Table 1:** Demographics and baseline characteristics of the subjects IOP: intraocular pressure; N: number; SD: standard deviation

Variable	Data
Total eyes (N)	46
Mean age in years±SD	73.0±8.5
Female (N, %)	28 (60.9%)
Male (N, %)	18 (39.1%)
Hispanic (N, %)	19 (41.3%)
White (N, %)	13 (26.1%)
Black or African American (N, %)	9 (19.6%)
Other/did not specify (N, %)	6 (13.4%)
Systemic hypertension (N, %)	25 (54.3%)
Diabetes mellitus (N, %)	13 (28.3%)
Mean axial length in mm±SD	23.3±0.9
Mean anterior chamber depth in mm±SD	2.8±0.3
Mean lens thickness in mm±SD	4.9±0.4
Previous selective laser trabeculoplasty (N, %)	6 (13.0%)
Previous laser peripheral iridotomy (N, %)	7 (15.2%)
Pre-op BCVA in LogMar±SD	0.4±0.4
Pre-op IOP in mm Hg±SD	19.3±6.0
Pre-op IOP-lowering medications (N±SD)	1.8±1.4

**Figure 1:**
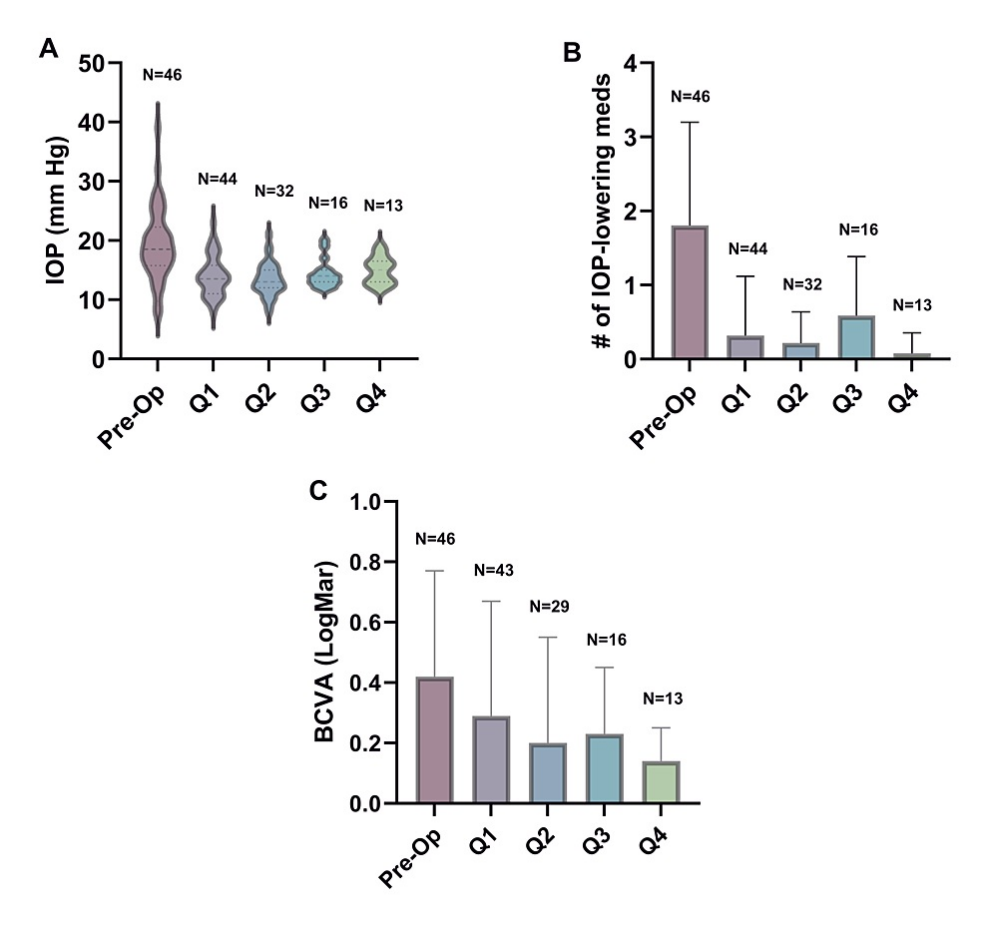
A graphic representation of the study outcomes. (A) A violin plot representing the intraocular pressure (IOP) changes over time. (B) A bar graph describing IOP-lowering medication dependency over time. (C) A bar graph describing best corrected visual acuity (BCVA) changes over time

All eyes included in the study had at least one follow-up within the first or second quartile. A total of 45 eyes (97.8%) were seen in the first quartile, 32 (69.6%) in the second, 16 (34.8%) in the third, and 13 (28.3%) in the fourth. The quartile outcomes for IOP measurements (Figure 1A), the number of IOP-lowering medications (Figure 1B), and BCVA (Figure 1C) are illustrated. Table 2 details the quartile mean, median, and standard deviation of these outcomes. A t-test comparing individual quartile outcomes with their respective preoperative values demonstrated statistically significant differences in all quartiles. A mixed-effects analysis showed a significant change over time for all three measurements.

**Table 2:** Pre- and postoperative measurements IOP: intraocular pressure; SD: standard deviation; N: number; Q: quartile

	Mean IOP±SD (median)	N	Mean # of meds±SD (median)	N	BCVA LogMar±SD (median)	N
Pre-op	19.3±6.0 (18.5)	46	1.8±1.4 (2)	46	0.4±0.4 (0.3)	46
Q1	14.0±3.3 (13.5)	44	0.3±0.8 (0)	44	0.3±0.4 (0.2)	43
Q1-pre-op p-value	<0.0001	-	<0.0001	-	<0.0001	-
Q2	13.4±2.8 (13)	32	0.2±0.4 (0)	32	0.2±0.4 (0.2)	29
Q2-pre-op p-value	<0.0001	-	<0.0001	-	0.0002	-
Q3	14.4±2.4 (14)	16	0.6±0.8 (0)	16	0.2±0.2 (0.2)	16
Q3-pre-op p-value	0.0003	-	0.0001	-	0.06	-
Q4	14.9±2.9 (15)	13	0.1±0.3 (0)	13	0.1±0.1 (0.18)	13
Q4-pre-op p-value	0.01	-	0.0003	-	0.004	-
Mixed-effects analysis p-value	<0.0001	-	<0.0001	-	0.0013	-

All adverse events were documented, regardless of whether they were related to the procedure (Table 3). Most of these events (17 of 18) had a transient and/or relatively mild nature, with one eye developing a postoperative retinal tear. The most frequent adverse event observed was posterior capsule opacification (19.6%).

**Table 3:** Intraoperative and postoperative adverse events IOP: intraocular pressure; N: number

Adverse event	N (%)
Posterior capsule opacity	9 (19.6%)
Mild anterior chamber inflammation	3 (6.5%)
IOP spike (ranged 38-45 mm Hg)	2 (4.4%)
Hyphema	1 (2.2%)
Retinal tear	1 (2.2%)
Transillumination defect	1 (2.2%)
Anterior capsule rent	1 (2.2%)

Only one subject in our sample was identified as a non-responder based on our criteria. The patient was an 83-year-old African-American female with a past medical history of hypertension and diabetes mellitus who preoperatively had an IOP of 18 mm Hg and took three IOP-lowering medications. By the third quartile (last follow-up on record), the patient had an IOP of 20 mm Hg and was back on three medications, which accounts for the slightly elevated Q3 medication mean (0.6±0.8; N=16).

## Discussion

This single-surgeon study is the first to provide insight into the real-world use of canaloplasty and trabeculotomy in conjunction with cataract surgery in patients with PACG. We aimed to describe a population that should not be excluded from consideration for this procedure, as it is currently off-label. The observed benefits from this intervention include significant improvements in IOP, lower postoperative medication burden at all follow-up visits (Figures 1A, 1B), and surgical resolution of closed angle anatomy. The latter is of particular importance in patients without prior LPIs, as it eliminates the risk of future acute angle closure. Notably, all the patients in our study had visually significant cataracts and had an objective improvement in vision after surgery (Figure 1C). We did not perform clear lens extraction in this group.

Although it has been shown that cataract removal alone reduces IOP in PACG (Table 4), glaucoma may persist post-phacoemulsification due to compromised outflow at the TM and SC. In PACG, this may stem from prolonged TM dysfunction caused by blood-aqueous barrier impairment, TM mitochondrial damage leading to fibrosis, SC endothelial aberrations resulting in narrowed collector canals, and obstruction from pigment granules and TM cells [7].

**Table 4:** Summary of studies investigating minimally invasive glaucoma surgery +/- phacoemulsification procedures and standalone phacoemulsification for primary angle-closure glaucoma GATT: gonioscopy-assisted transluminal trabeculotomy; GSL: goniosynechialysis; GT: goniotomy; KDB: Kahook dual blade; MIGS: minimally invasive glaucoma surgery; MVR: microvitreoretinal; N: number of eyes; NA: not applicable; PACG: primary angle-closure glaucoma; PAS: peripheral anterior synechia; PEI: phacoemulsification lens extraction with intraocular lens implantation; Q: quartile; SD: standard deviation; TLT: transscleral laser therapy; TMH: Tanito microhook; UCP: ultrasound ciliary plasty

Study	Country	Procedure	Follow-up (months)	Mean baseline IOP in mmHg (N)	Mean post-op IOP in mm Hg (N)	Mean baseline # of Meds (N)	Mean post-op # of Meds (N)
Dorairaj et al., 2019 [11]	Vietnam	PEI+GSL+GT (KDB)	24	25.5±0.7 (42)	13.5±0.4 (42)	2.3±0.8 (42)	0.6±0.2 (42)
Hernstadt et al., 2019 [12]	Singapore	PEI+iStent	12	24.6±3.4 (unmedicated) 17.5±3.82 (medicated) (37)	14.8±3.9 (37)	1.5±0.8 (37)	0.1±0.5 (37)
Husain et al., 2019 [13]	Singapore, Vietnam, Thailand, Hong Kong	PEI only	12	22.3±8.5 (40)	12.5±2.7 (40)	2.2±0.8 (40)	0.5±0.9 (40)
Chen et al., 2020 [14]	Singapore	PEI+iStent/PEI	12	18.6±3.1 (16 PEI+iStent); 17.5±3.1 (16 PEI)	14.7±3.1 (16 PEI+iStent); 15.0±2.5 (16 PEI)	1.5 (16 PEI+iStent) 2.0 (16 PEI)	0.3±0.7 (16 PEI+iStent); 0.8±1.0 (16 PEI)
Nguyen et al., 2020 [15]	USA	MicroPulse TLT	12	25.1±5.3 (95, 15 PACG)	17.5±5.1 (95, 15 PACG)	3.0±1.1 (95, 15 PACG)	1.4±1.0 (95, 15 PACG)
Chira-Adisai et al., 2021 [16]	Japan	PEI+GSL+GATT/GSL+GATT	24	21.8±5.4 (46)	15.1±4.4 (46)	3.5±1.4 (46)	1.5±0.5 (46)
Gupta et al., 2021 [17]	India	PEI+GSL+GT/PEI+GT (MVR blade)	12	21.4±6.6 (46)	14.2±3.7 (46)	3.3±1.0 (46)	1.5±1.2 (46)
Helmy et al., 2021 [18]	Egypt	PEI only	240	22.2±2.08 (102)	14.1±2.1 (102)	2.7±0.5 (102)	0.5±0.8 (102)
Salimi et al., 2021 [19]	Canada	PEI+iStent /PEI	12	17.6±3.2 (79 PEI+iStent); 16.8±3.1 (79 PEI)	12.9±2.3 (79 PEI+iStent); 14.6±2.5 (79 PEI)	2.2±1.2 (79 PEI+iStent); 1.8±1.3 (79 PEI)	1.6±1.4 (79 PEI+iStent); 1.8±1.3 (79 PEI)
Sharkawi et al., 2021 [20]	Japan	PEI+GATT	24	21.4±7.4 (103)	12.1±2.4 (103)	2.5±1.1 (103)	0.8±1.2 (103)
Shokoohi-Rad et al., 2021 [21]	Iran	PEI+GSL+GT; PEI+GSL	6	20.7±5.5 (32); 19.9±4.5 (31)	13.7±2.4 (32); 15.3±2.8 (31)	1.3±0.8 (32); 1.3±0.94 (31)	0.4±0.6 (32); 0.7±0.7 (31)
Tanito et al., 2021 [22]	Japan	PEI+GSL+GT/PEI+GT (TMH)	36	20.2±7.0 (560)	13.9±4.5 (560)	2.8±1.1 (560)	2.5±1.0 (560)
Wang et al., 2021 [23]	China	PEI+GSL+Trabectome	12	22.1±6.6 (22)	15.1±3.4 (22)	2.7±1.2 (22)	0.8±0.7 (22)
Wanichwecharungruang et al., 2021 [24]	Thailand	PEI only	12	16.0±2.2 (84)	14.0±2.0 (84)	2.0±0.7 (84)	1±0.6 (84)
Fontana et al., 2022 [25]	Italy	GATT+GSL/GATT	12	30.3±4.2 (15)	15.2±2.1 (15)	3.5±0.5 (15)	0.3±0.6 (15)
Senthil et al., 2022 [26]	India	PEI only	30	15.2±3.7 (33)	14.9±2.9 (33)	1.6±0.8 (33)	0.5±0.7 (33)
Tan et al., 2023 [27]	China	PEI+GSL+GT (KDB)	12	23.4±8.1 (300)	16.6±3.9 (300)	2.6±1.3 (300)	0.4±0.9 (300)

When glaucoma patients require cataract surgery, minimally invasive glaucoma surgery can be performed simultaneously to treat their condition with minimal additional risk (Table 4). It may be unethical and more costly to subject these patients to a separate surgery if it can be avoided by performing a concurrent glaucoma procedure. It may decrease the risk of escalating to more invasive approaches such as trabeculectomy or cataract surgery with tube shunts. Furthermore, we hypothesize that targeting multiple sources of aqueous outflow resistance [10] (TM, SC, and collector channels) with the OMNI surgical system yields greater IOP reduction effects than standalone phacoemulsification.

Adverse events were similar to those experienced by patients with open-angle glaucoma phenotypes [10,28]. PCO was the most frequently reported adverse event (19.6%). This is within the established range of posterior capsular opacification found after standard phacoemulsification with intraocular lens without concurrent glaucoma surgery [29].

There are legitimate concerns about the costs and increased risks of adding MIGS to cataract surgery. However, the promising results, the likelihood of getting off drops, and the low complication rates observed may alleviate this concern. Further investigation and a cost analysis of these procedures are warranted here.

Aside from the therapeutic benefits we have seen in this group, there is a high incidence of hyperopia in these patients. Our threshold was 20/40 BCVA (0.30 LogMar) or worse, attributable to cataracts. Consequently, there was often a dramatic improvement in postoperative uncorrected distance visual acuity, even in the case of mild cataracts. We also noticed a continued improvement in visual acuity months after surgery (Figure 1C). This is consistent with our experience that the ocular surface health improved over time with the withdrawal of glaucoma drops. While these results are encouraging, we did have one non-responder. Non-responders are common in all varieties of glaucoma, warranting further investigation into factors underlying nonresponse or decreased response to these interventions. As more data becomes available, it would be helpful to predict which patients are less likely to respond to this surgery.

A limitation of the study was the need for a washout period. Our study was retrospective and therefore patient care reflects standard medical practice, in which pre-surgical washouts are not routine. The possibility of overmedication preoperatively does exist due to the referral-based practice setting. In patients who were overmedicated preoperatively, the decrease in postoperative medications would be exaggerated. The overall trend of improvement in IOP with a concurrent reduction in medication burden postoperatively is encouraging, though. It suggests the topical medications weren’t prescribed needlessly before surgery and highlights the potency of the intervention. Our sample was not stratified into glaucoma severity, and although we included some advanced glaucoma patients, we recommend extra caution in treating this group. We have achieved low target IOPs in advanced angle closure patients with phacoemulsification/goniotomy/canaloplasty alone. Out of caution, we tended to keep advanced angle closure glaucoma patients on drops for longer in the immediate postoperative period. Another notable group was the patients who already had an iridotomy. It would be interesting to investigate this population further. They presumably lack the intermittent angle closure element that a relative pupil block can exacerbate, so their postoperative improvement might not be as dramatic. Alternatively, there might be patients who have more of an iris plateau configuration, given their angles were persistently closed after iridotomy - this could conceivably temper their response to this surgery, but they might also be patients who respond well to pilocarpine’s effect in drawing the plateau iris out of the crowded angle-even post-surgery. Our preoperative imaging of the angle was limited to gonioscopy, but it would have been informative to compare different angle architectures and their response to this therapy pre- and postoperatively.

This is a surgery that could comfortably replace trabeculectomy for many patients with mild to moderate chronic angle-closure glaucoma. It would be informative to consider this combination of surgeries for patients with clear lenses: How would the canaloplasty/goniotomy component change the risk/benefit calculus over and above what has already been established by the EAGLE trial [30]? We suggest that this combination should be considered a safer and more effective alternative to trabeculectomy, phacoemulsification/trabeculectomy, or phacoemulsification/tube surgery in mild-to-moderate angle closure patients.

As a retrospective review in a clinical setting, we recognize the limitations of this study, such as the absence of a control group, the relatively small sample size, and the limited follow-up data. We hope, though, that this “real-world data” can provide some insights to others considering this procedure for closed-angle glaucoma. More research is needed, but based on our experience outlined here, we can advocate for this approach as a safe and effective alternative to more traditional interventions for closed-angle glaucoma.

## Conclusions

Combining the OMNI surgical system with phacoemulsification led to substantial reductions in mean IOP and the number of IOP-lowering medications when compared to baseline measurements. Due to limited statistical power and study limitations, findings should be interpreted cautiously.
